# Supplementary material for: Removal of Oily Contaminants from Water by Using the Hydrophobic Ag Nanoparticles Incorporated Dopamine Modified Cellulose Foam
Source: Polymers (Basel). 2021 Sep 18;13(18):3163. doi: 10.3390/polym13183163 (PMC8471367; doi:10.3390/polym13183163)
Supplement: Supplementary file 1 [file polymers-13-03163-s001.zip › Suplementray Materials.pdf]

Supporting Information for

Removal of oily contaminants from water by using the hydrophobic nanoparticle incorporated dopamine modified cellulose foam

Nadeem Baig*[1] and Irshad Kammakakam*[2]

[1]Interdisciplinary Research Center for Membranes and Water Security, King Fahd University of Petroleum and Minerals, Dhahran 31261, Saudi Arabia

[2]Department of Chemical & Biological Engineering, University of Alabama, Tuscaloosa, Alabama 35487-0203, USA

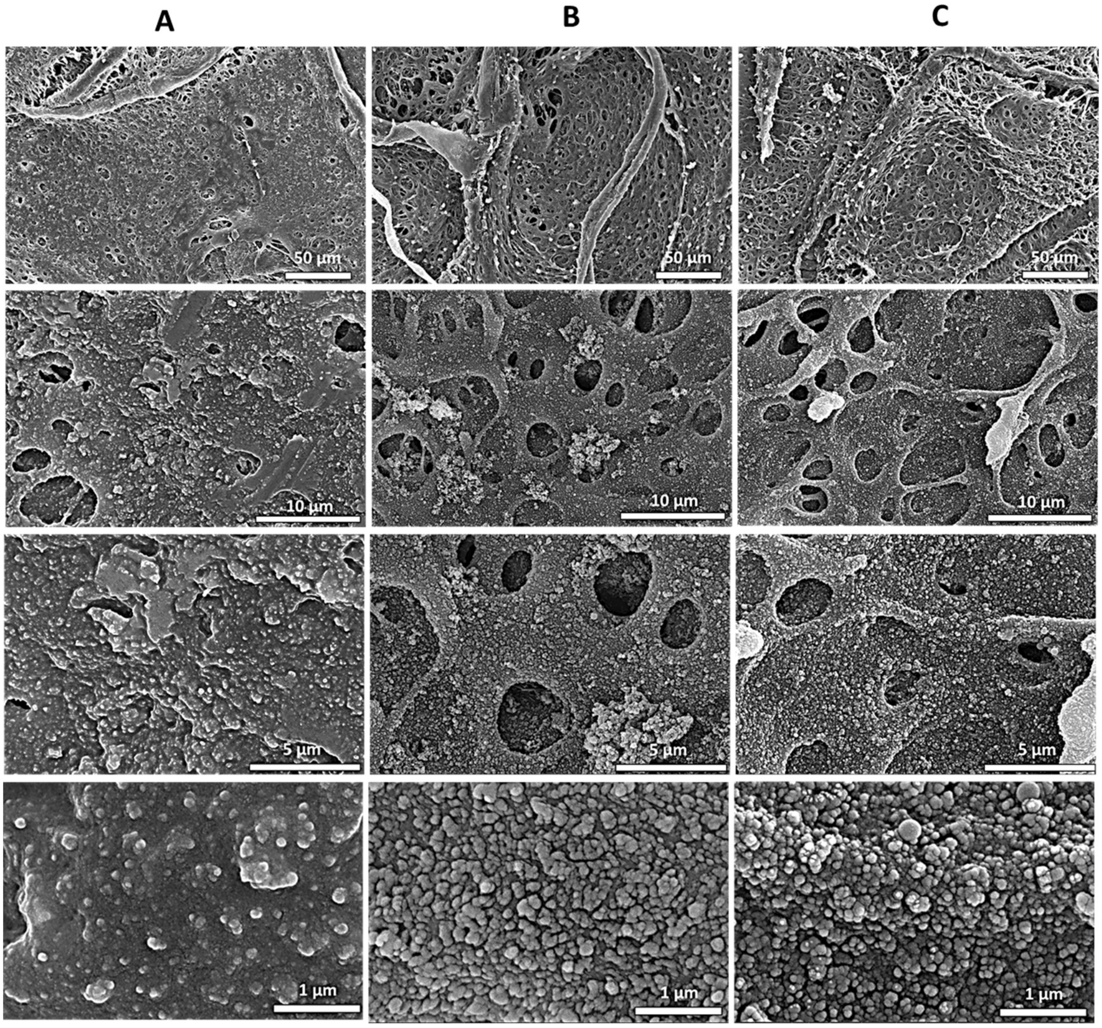

Fig. S1 SEM images of (A) Ag-DA-Cell-F, (B) C1s-Ag-DA-Cell-F, (C) C4s-Ag-DA-Cell-F

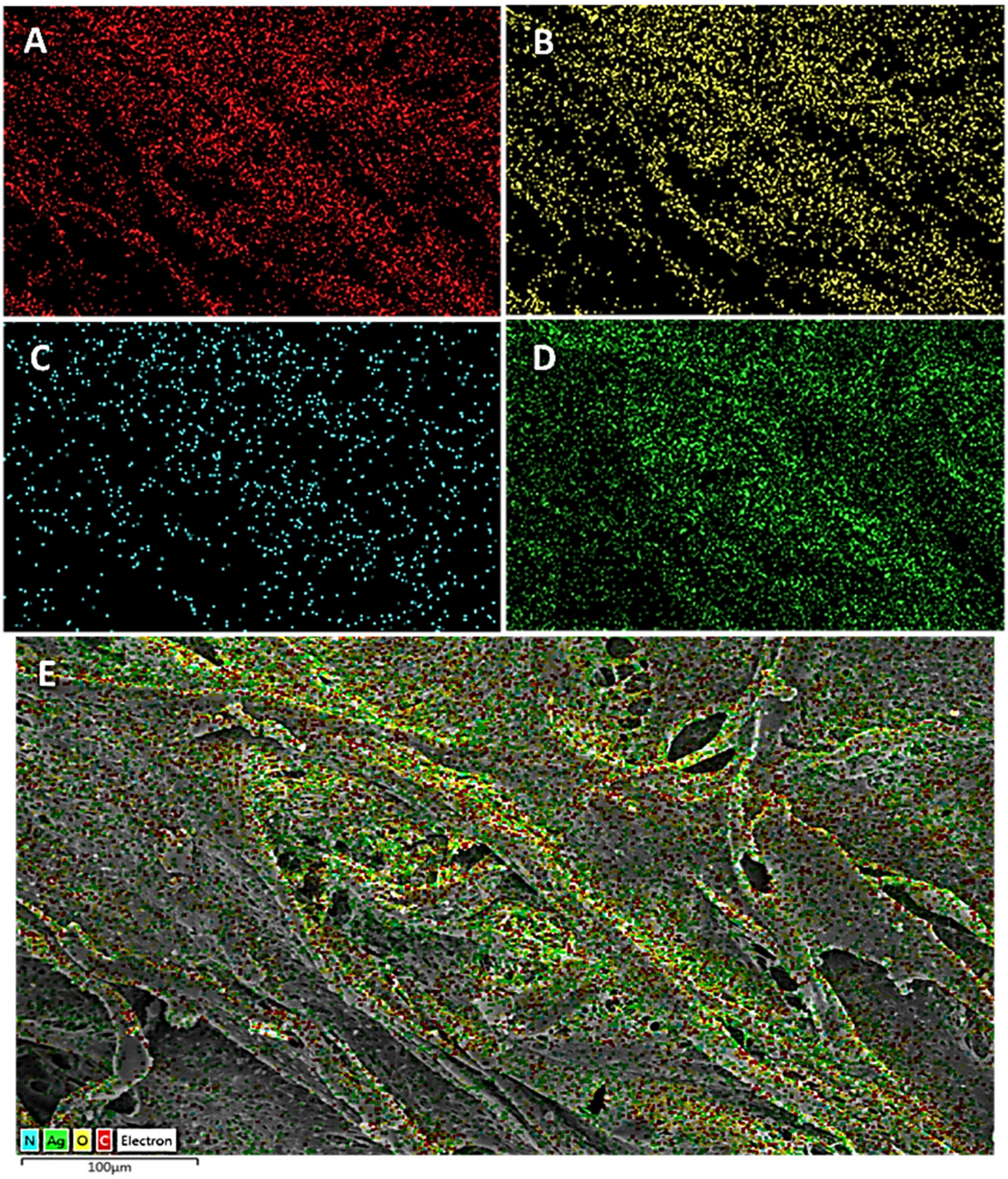

Fig. S2 Elemental mapping of Ag-DA-Cell-F (A) C, (B) O, (C) N, (D) Ag and (E) EDS layered image

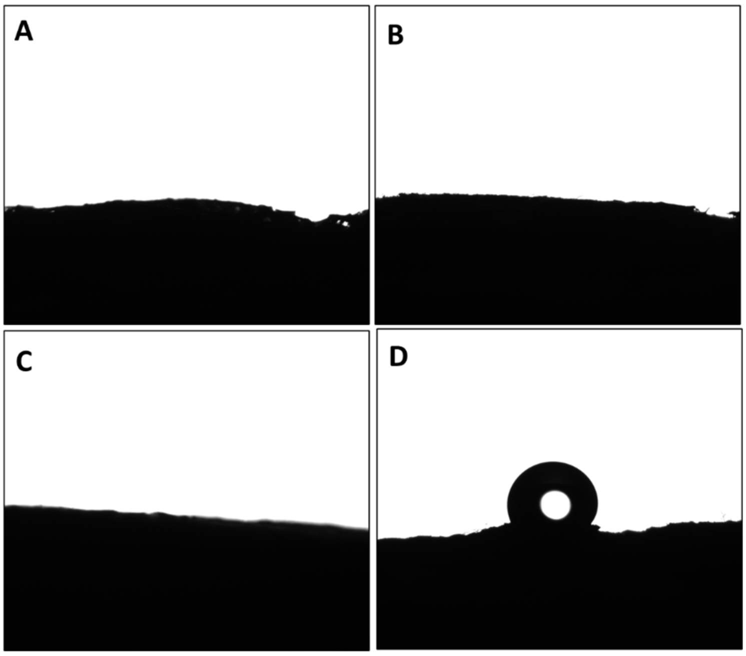

Fig. S3 Water contact angle on (A) DA-Cell-F, (B) C1s-Ag-DA-Cell-F, (C) C4s-Ag-DA-Cell-F, (D) C8s-Ag-DA-Cell-F
